# Diagnostic Accuracy of GPT-5 in Detecting and Localizing Acute Ischemic Stroke on Isolated Diffusion-Weighted Imaging: A Retrospective Case–Control Study

**DOI:** 10.3390/jcm15197716

**Published:** 2026-10-06

**Authors:** Emre Gökçen, Mikail Kuşdoğan, Mustafa Fatih Erkoc, Levent Albayrak, Güneş Seda Albayrak, Deniz Tanrıverdi

**Affiliations:** 1 Department of Emergency Medicine, Faculty of Medicine, Yozgat Bozok University, 66100 Yozgat, Turkey; 2 Department of Radiology, Faculty of Medicine, Yozgat Bozok University, 66100 Yozgat, Turkey; mustafafatih.erkoc@bozok.edu.tr; 3 Department of Neurology, Faculty of Medicine, Yozgat Bozok University, 66100 Yozgat, Turkey; 4 Department of Emergency Medicine, Dr. Nevruz Erez State Hospital, 76000 Iğdır, Turkey

**Keywords:** acute ischemic stroke, diffusion-weighted imaging, large language model, GPT-5, artificial intelligence, vascular territory, emergency radiology

## Abstract

**Background/Objectives:** Multimodal large language models are emerging tools for medical image interpretation, but their performance on isolated diffusion-weighted imaging (DWI) for acute ischemic stroke remains largely unexplored. We evaluated GPT-5 for stroke detection and vascular territory classification on preselected representative DWI images using a structured two-step prompting protocol. **Methods:** This single-center, retrospective, balanced case–control study included 220 cases (110 stroke patients and 110 stroke-negative controls). Four representative axial DWI slices per case were provided without apparent diffusion coefficient maps. Step 1 queried stroke presence and vascular territory; Step 2 queried the middle cerebral artery (MCA) segment-associated territory (M1–M4) following an MCA response. The reference standard was radiologist–neurologist consensus based on full multimodal magnetic resonance imaging, blinded to GPT-5 outputs. Diagnostic performance metrics and Cohen’s kappa (κ) were calculated. **Results:** Stroke detection showed a sensitivity of 100% (95% confidence interval [CI], 96.6–100%), specificity of 83.6% (95% CI, 75.6–89.4%), accuracy of 91.8%, and κ = 0.836. Accuracy was 93.6% for anterior/posterior circulation (κ = 0.838) and 90.9% for vascular territory (κ = 0.799). Conditional MCA segment-associated territory accuracy was 71.6% (53/74; κ = 0.602); end-to-end accuracy was 66.3% (53/80). Exploratory analysis of the 18 false-positive cases identified four overlapping error categories: artifactual and physiologic signal misinterpretation (72.2%), chronic lesion misclassification (27.8%), terminological inconsistency or unsupported report statements (77.8%), and etiological scenarios or unhedged definitive diagnoses (38.9%). **Conclusions:** GPT-5 achieved high sensitivity and a specificity of 83.6% for stroke classification on preselected representative DWI images using a vascular territory-based diagnostic framework. However, the 16.4% false-positive rate indicates that positive findings require radiologist confirmation. These findings support further evaluation of GPT-5 as a potential clinician-assisted image-interpretation tool but do not establish a clinical decision-support role.

## 1. Introduction

Acute ischemic stroke remains a leading cause of mortality and long-term disability worldwide [1], and early and accurate diagnosis is critical for timely therapeutic intervention [2]. Diffusion-weighted magnetic resonance imaging (DWI) is the cornerstone of acute stroke diagnosis, offering high sensitivity for cytotoxic edema within minutes of ischemic onset [1,3,4]. In emergency settings where rapid image interpretation is essential, diagnostic support tools that facilitate timely and accurate DWI analysis could provide meaningful clinical utility, particularly where subspecialty radiology support is delayed or unavailable. Recent efforts have accordingly explored artificial intelligence (AI)-based approaches for automated DWI interpretation [4,5].

Large language models (LLMs) with multimodal vision capabilities have recently emerged as promising tools for medical image interpretation, demonstrating encouraging performance across a range of radiological tasks [6,7]. However, their application to DWI-based stroke diagnosis remains limited, with only a small number of studies published to date [3,5]. Existing reports have shown substantial variability in diagnostic performance, even among studies evaluating closely related LLMs [3,5,8,9]. This variability may reflect differences in model generation, prompting strategy, imaging input, and study design, although their relative contributions remain unclear. The performance of a contemporary general-purpose LLM on isolated DWI, using clinically defined vascular territories as the diagnostic framework, and the characteristic errors associated with this approach have not yet been systematically evaluated.

The present study evaluated GPT-5 on preselected representative DWI images using a structured, vascular territory-based two-step prompting protocol. A vascular territory-based framework was adopted as an alternative to the hemisphere- and lobe-based localization strategies used in previous studies [3,5]. The protocol queried the major vascular territories (middle cerebral artery [MCA], posterior inferior cerebellar artery [PICA], anterior cerebral artery [ACA], and posterior cerebral artery [PCA]) and, for MCA infarctions, further localized lesions to M1–M4 segment-associated territories. This design was intended to evaluate GPT-5 performance across clinically relevant levels of anatomical localization and to characterize the diagnostic error patterns associated with this approach.

## 2. Methods

### 2.1. Study Design and Patient Selection

This retrospective, balanced case–control study was conducted at the Faculty of Medicine, Yozgat Bozok University. The study protocol was approved by the Non-Interventional Clinical Research Ethics Committee of Yozgat Bozok University on 4 December 2024 (approval number 2024-GOKAEK-2414_2024.12.04_211) and was conducted in accordance with the Declaration of Helsinki. Informed consent was waived owing to the retrospective design.

Between January 2021 and December 2024, 1248 consecutive patients aged ≥18 years who presented to the emergency department with acute focal neurological deficits suggestive of stroke and underwent a standardized cranial magnetic resonance imaging (MRI) protocol including DWI within 24 h of symptom onset were screened. Eligibility was assessed by two emergency physicians in consensus against predefined clinical and imaging criteria.

Overall, 258 patients were excluded according to predefined criteria: suboptimal image quality due to severe motion artifacts (*n* = 114); non-ischemic intracranial pathology that could confound DWI interpretation, including primary brain tumors, metastases, or vascular malformations (*n* = 62); prior large-vessel territory infarction in the same anatomical region (*n* = 54); and non-diagnostic or incomplete MRI examinations (*n* = 28). After application of the predefined eligibility and exclusion criteria, 990 examinations remained eligible.

Acute ischemic stroke status for each eligible examination was determined according to the reference standard (Section 2.4), which classified 110 of the 990 eligible examinations as stroke-positive and 880 as stroke-negative.

**Figure 1:**
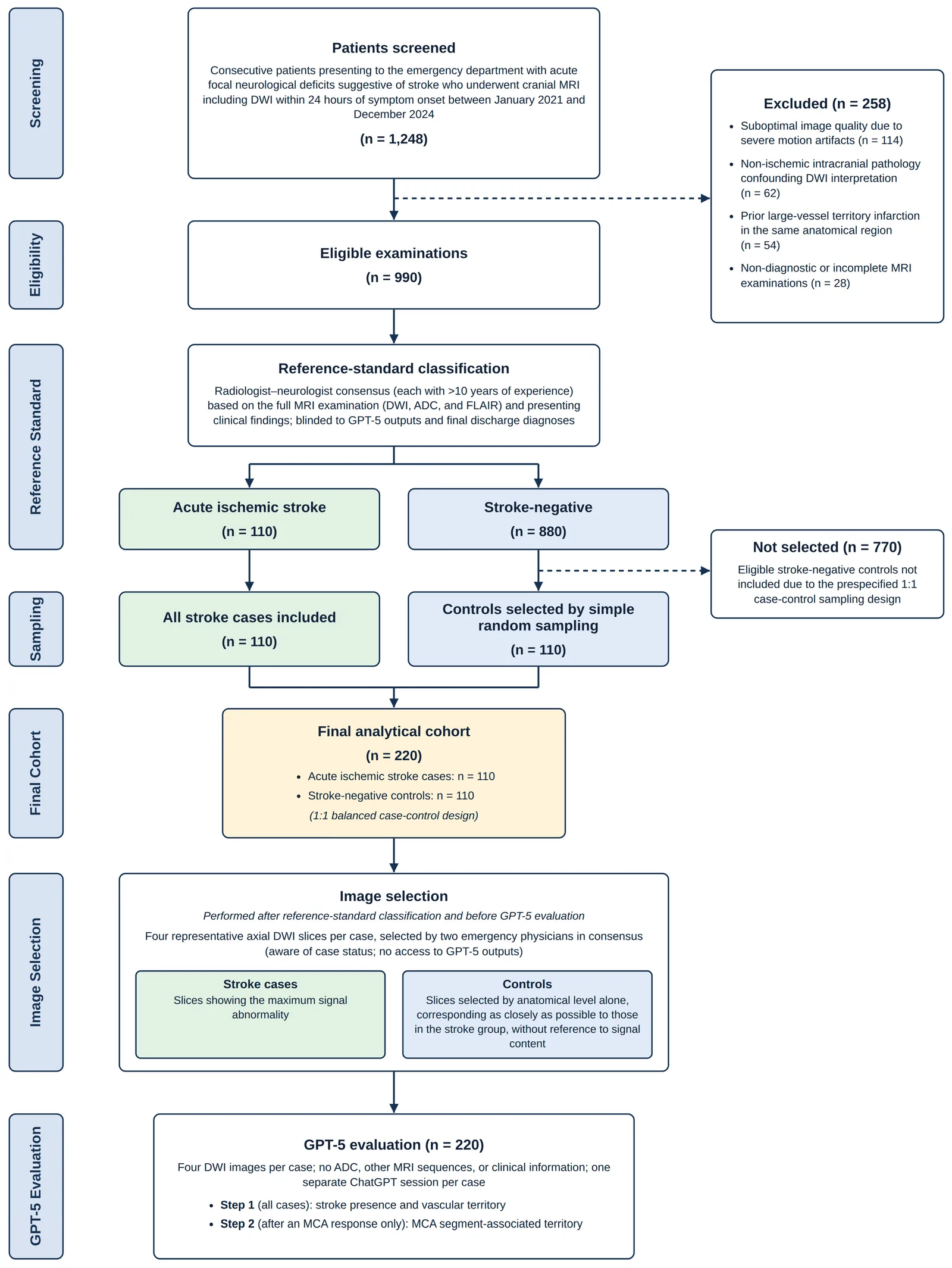
Study flow: patient selection, reference-standard classification, image selection, and GPT-5 evaluation. Of the 1248 patients screened, 258 were excluded according to predefined eligibility criteria. Among the 990 eligible examinations classified by the reference standard, all 110 acute ischemic stroke cases were included, and 110 stroke-negative controls were randomly selected from the eligible control pool (*n* = 880) to establish a balanced 1:1 case–control cohort. The final analytical cohort consisted of 220 patients. Image selection was performed after reference-standard classification and before GPT-5 evaluation (Section 2.2), and all 220 cases were then evaluated by GPT-5 using the two-step prompting protocol (Section 2.3). MRI, magnetic resonance imaging; DWI, diffusion-weighted imaging; ADC, apparent diffusion coefficient; FLAIR, fluid-attenuated inversion recovery; MCA, middle cerebral artery.

All 110 eligible stroke cases were included. The control group was drawn from the same consecutive emergency department population as the stroke cases. All control patients had presented with acute focal neurological symptoms prompting clinical evaluation for suspected stroke and had undergone the same standardized MRI pathway. Stroke-negative status was established by the radiologist–neurologist reference standard using the available multimodal MRI and presenting clinical information. From the 880 eligible stroke-negative examinations, 110 controls were selected by simple random sampling. The stroke group comprised acute ischemic infarctions across multiple vascular territories (MCA, ACA, PCA, and PICA), whereas the control group consisted of patients without acute ischemic lesions on multimodal MRI, some of whom had non-acute imaging findings such as chronic microangiopathy. After completion of the reference-standard classification, the clinical diagnoses recorded in the hospital records of the control patients were retrieved and grouped into diagnostic categories for descriptive purposes. The final analytical cohort therefore comprised 220 patients (110 stroke patients and 110 stroke-negative controls). The study flow is summarized in Figure 1.

### 2.2. Image Acquisition and Preparation

All DWI examinations were performed using a 1.5-T MRI scanner (Philips Ingenia; Philips Healthcare, Best, Netherlands) with a slice thickness of 5 mm and b-values of 0 and 1000 s/mm^2^.

For each stroke case, four representative axial DWI slices demonstrating the area of maximum signal abnormality were selected according to a predefined image-selection protocol by two emergency physicians in consensus. For control cases, four slices were selected on the basis of anatomical level alone, corresponding as closely as possible to the anatomical levels represented in the stroke group, without reference to signal content and avoiding preferential selection of artifact-free images, to provide comparable anatomical sampling between groups. The purpose of this procedure was to provide GPT-5 with a standardized set of representative DWI images rather than the complete DWI series.

The image-selection procedure was performed after establishment of the reference-standard classification and before GPT-5 evaluation (Figure 1). Accordingly, the two emergency physicians were not blinded to the reference-standard case status (stroke versus control) during slice selection, although they had no access to GPT-5 outputs. Because slice selection in stroke cases was based on the predefined criterion of maximum visible signal abnormality, the procedure was designed to evaluate GPT-5’s ability to detect and localize acute ischemic abnormalities when diagnostically representative isolated DWI images were presented, rather than to assess autonomous lesion search across the complete DWI series.

Apparent diffusion coefficient (ADC) maps were intentionally excluded from the evaluation protocol because the objective of this study was to characterize GPT-5 performance using isolated DWI rather than to maximize diagnostic accuracy through multimodal image interpretation. Multimodal inputs combining DWI and ADC provide cross-sequence confirmation; however, restricting the model input to a single sequence allowed us to isolate performance attributable to DWI interpretation. Including ADC would have introduced an additional source of diagnostic information, making it more difficult to determine whether model decisions were based primarily on the DWI findings or on cross-sequence confirmation. Given the limited interpretability of the internal decision processes of current multimodal LLMs, this distinction was considered methodologically important. Accordingly, the isolated-DWI design provided a controlled framework for characterizing both diagnostic performance and error patterns associated with DWI interpretation alone. However, this experimental condition differs from routine clinical practice, in which DWI is interpreted together with ADC to confirm true diffusion restriction and to distinguish it from T2 shine-through and susceptibility- or pulsation-related artifacts.

Images were exported from Digital Imaging and Communications in Medicine (DICOM) files and converted to JPEG format without adjustment of brightness or contrast. The b = 1000 s/mm^2^ images were used as model input, and each case was uploaded as four separate JPEG files (1325 × 615 pixels each). All images were anonymized before upload, with patient identifiers and DICOM metadata removed. Image filenames were standardized to eliminate contextual cues that might influence model interpretation.

### 2.3. AI Model and Prompting Protocol

GPT-5 (OpenAI, San Francisco, CA, USA; GPT-5 Thinking, accessed via ChatGPT) was used for all image evaluations. All image assessments were conducted between September 2025 and February 2026. All image evaluations were performed using this model through the ChatGPT interface, without investigator-initiated changes to the model selection during the study. The application programming interface (API) was not used, and backend build identifiers and sampling parameters not exposed by the interface were unavailable for recording. A standardized two-step sequential prompting protocol was developed prior to data collection and applied uniformly across all cases.

**Figure 2:**
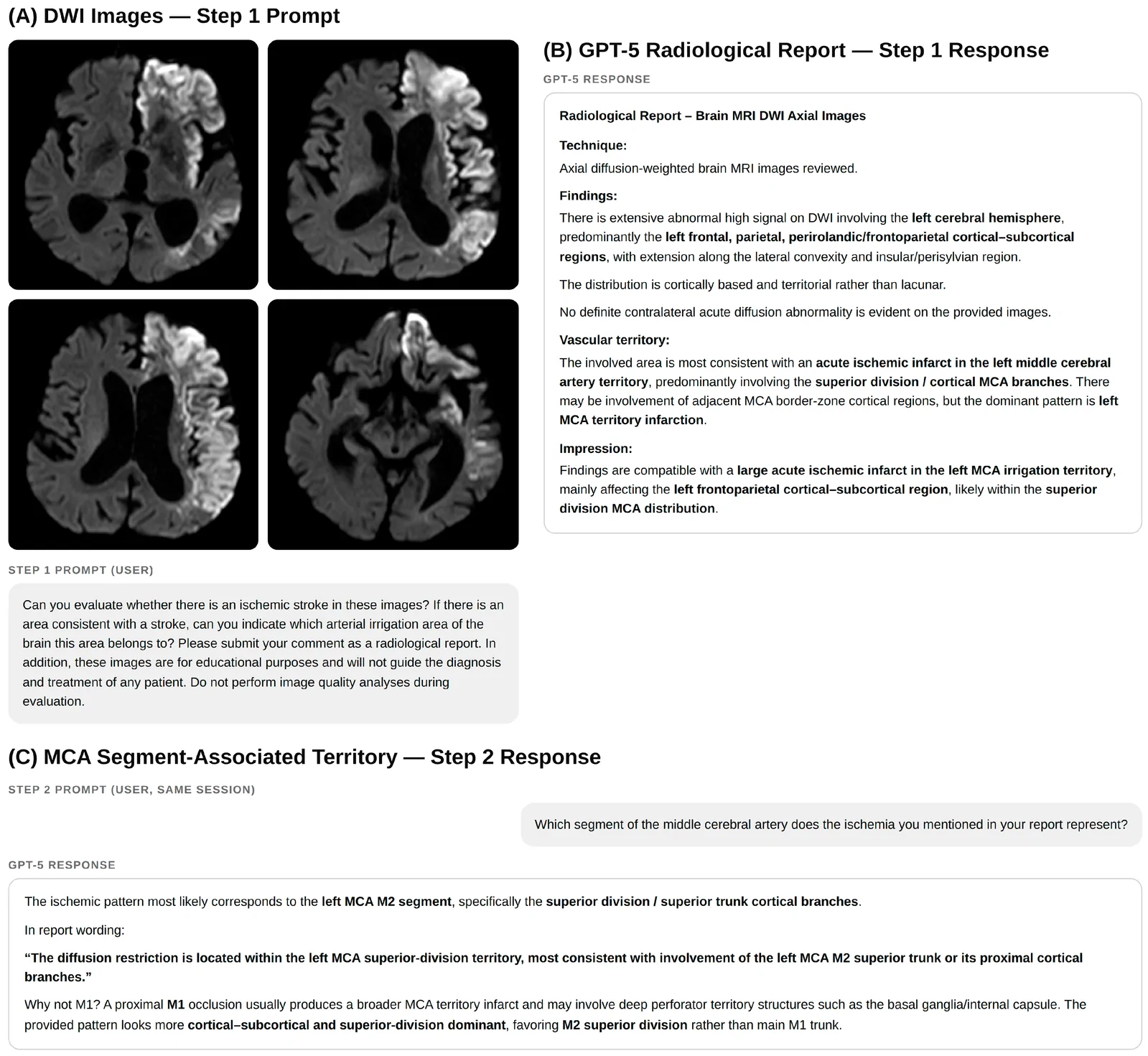
Representative true-positive case demonstrating the GPT-5 two-step sequential prompting protocol. (**A**) Four axial DWI slices uploaded simultaneously with the standardized Step 1 prompt. (**B**) GPT-5 radiological report identifying acute ischemic infarction in the left MCA territory, superior division. (**C**) GPT-5 response to the Step 2 prompt identifying MCA M2 segment-associated territory. The reference standard confirmed acute ischemic stroke in the left MCA M2 segment-associated territory. Prompt and response texts are reproduced verbatim from the original GPT-5 session and re-typeset for legibility; bold formatting reflects the original model output. Panel labels were added and interface elements were removed. Panel A reproduces the four axial DWI images submitted to GPT-5 from the original image files; only empty background was cropped, and no other image processing was applied.

In the first step (Step 1), four representative DWI slices were uploaded simultaneously. No ADC or fluid-attenuated inversion recovery (FLAIR) images, other MRI sequences, or clinical information were provided to GPT-5. The following prompt was presented (Figure 2):

“Can you evaluate whether there is an ischemic stroke in these images? If there is an area consistent with a stroke, can you indicate which arterial irrigation area of the brain this area belongs to? Please submit your comment as a radiological report. In addition, these images are for educational purposes and will not guide the diagnosis and treatment of any patient. Do not perform image quality analyses during evaluation.”

The requirement for the output to be formatted as a radiological report was a deliberate methodological choice intended to generate structured, radiology-style output rather than simple binary classifications. Beyond this primary rationale, the structured reporting requirement also provided an opportunity to evaluate how the model reconciles domain-specific reporting expectations with the limited information available from isolated DWI images.

When the model identified MCA territory involvement in the first step, a second prompt (Step 2) was administered within the same session:

“Which segment of the middle cerebral artery does the ischemia you mentioned in your report represent?”

The second step was therefore applied only to cases with an MCA response. Each case was evaluated once in a separate ChatGPT session using an identical prompting protocol to prevent carry-over effects from prior interactions. Model responses were compared against the pre-established radiologist–neurologist consensus reference standard using predefined classification criteria: a response was classified as correct only if the identified territory or segment-associated territory exactly matched the reference standard. Partially correct or ambiguous responses were classified as incorrect.

During prompt development, the finalized prompting protocol was independently repeated in a separate 34-case image set that was not included in the final study cohort. The same four-image inputs and final prompts were used in separate ChatGPT sessions with the same GPT-5 model to explore response stability.

### 2.4. Reference Standard

The reference standard was established independently by a radiologist and a neurologist with more than 10 years of experience in stroke imaging and stroke management, respectively. Both experts reviewed the complete available MRI examination, including DWI, ADC, and FLAIR sequences, together with the presenting clinical findings. They remained blinded to GPT-5 outputs and to the final clinical discharge diagnosis throughout the reference-standard assessment. In cases of disagreement, the images and relevant clinical information were re-evaluated jointly until consensus was reached. Individual pre-consensus assessments were not retained; therefore, inter-reader agreement for the reference standard could not be quantified.

The reference standard was recorded hierarchically at three levels: binary stroke presence, primary vascular territory (MCA, ACA, PCA, or PICA), and MCA segment-associated territory (M1–M4).

The M1–M4 labels follow the conventional nomenclature of the MCA segments; in this study, they denoted the presumed segment-associated territory, assigned according to the predominant distribution of the ischemic lesion on MRI, rather than the arterial segment itself or a directly confirmed occlusion site. Angiographic imaging was not used to define the M1–M4 reference labels. When an infarction extended across more than one segment-associated territory, a single reference label was assigned by radiologist–neurologist consensus according to the predominant lesion distribution. The M2, M3, and M4 segment-associated territories were treated as mutually exclusive categories as a methodological simplification to enable single-label anatomical classification and categorical agreement analysis.

### 2.5. Statistical Analysis

Statistical analyses were performed using IBM SPSS Statistics for Windows, Version 24.0 (IBM Corp., Armonk, NY, USA). Sensitivity, specificity, positive predictive value (PPV), negative predictive value (NPV), and overall accuracy were calculated, with Wilson score 95% confidence intervals (CIs) for proportions. Because the study cohort was deliberately balanced at a 1:1 stroke-to-control ratio, PPV and NPV were regarded as cohort-specific descriptive measures rather than population-level predictive values. Agreement between GPT-5 classifications and the radiologist–neurologist consensus reference standard was assessed using Cohen’s kappa coefficient (κ). For multiclass vascular territory classification, a complete predicted-versus-reference confusion matrix was constructed, and class-specific performance was evaluated using a one-versus-rest approach. To account for class imbalance, macro-averaged recall was calculated as the unweighted mean of class-specific recalls; this measure is equivalent to multiclass balanced accuracy. Performance for the MCA segment-associated territory was evaluated both conditionally, among cases in which GPT-5 first correctly identified the MCA territory, and as end-to-end hierarchical accuracy across all 80 reference-standard MCA cases. The exploratory repeatability analysis was summarized using percentage agreement and Cohen’s κ for binary stroke classification; vascular territory concordance was reported descriptively among cases classified as stroke in both runs. Pearson chi-square tests were used to compare sex distribution between groups and to assess the association between GPT-5 classifications and the reference standard. Age was compared using the independent-samples *t*-test after confirming approximately normal distributions with the Shapiro–Wilk test. A *p* value < 0.05 was considered statistically significant.

### 2.6. False-Positive Error Analysis

After completion of the blinded reference-standard adjudication and the primary diagnostic analysis, the 18 false-positive cases were evaluated in a separate post hoc exploratory error analysis. Four non-mutually exclusive error categories were derived inductively by the investigator team (two emergency physicians, one radiologist, and one neurologist), and operational definitions and boundary rules were finalized before formal case-level coding. Two emergency physicians then independently coded all 18 cases using the predefined coding framework. Because the analysis was restricted to known false-positive cases, the coders were aware of false-positive status but were blinded to each other’s assignments and to the final clinical discharge diagnoses. Discrepant assignments were reviewed with the radiologist and neurologist and resolved by consensus. Individual pre-consensus assignments were not retained; therefore, inter-rater reliability could not be quantified. Case-level category and sub-code assignments are provided in Supplementary Tables S1 and S2. The category labels describe the observed content of the model output and do not imply a causal mechanism.

## 3. Results

### 3.1. Study Population

All 220 cases in the final cohort (110 stroke patients and 110 stroke-negative controls) were included in the primary analysis, with no exclusions. The study population comprised 132 males (60.0%) and 88 females (40.0%). The stroke group included 67 males (60.9%) and 43 females (39.1%), and the control group included 65 males (59.1%) and 45 females (40.9%), with no significant difference in sex distribution between groups (*p* = 0.783). The mean age was 70.8 ± 12.1 years in the stroke group and 68.3 ± 11.9 years in the control group, with no significant difference (*p* = 0.122). The clinical diagnoses recorded for the control group were transient ischemic attack (*n* = 38, 34.5%), headache disorders (*n* = 21, 19.1%), seizure or post-ictal state (*n* = 13, 11.8%), vestibular disorders (*n* = 12, 10.9%), toxic or metabolic encephalopathy (*n* = 9, 8.2%), functional neurological disorders (*n* = 4, 3.6%), and other or undetermined diagnoses (*n* = 13, 11.8%).

### 3.2. Diagnostic Performance in Stroke Detection

GPT-5 correctly classified all 110 stroke cases, yielding an observed sensitivity of 100% (110/110; 95% CI, 96.6–100%). Eighteen stroke-negative control patients were incorrectly identified as stroke (false positives), yielding a specificity of 83.6% (95% CI, 75.6–89.4%). Overall diagnostic accuracy was 91.8% (95% CI, 87.4–94.8%). Within the balanced study cohort, the PPV was 85.9% (95% CI, 78.9–90.9%) and the NPV was 100% (95% CI, 96.0–100%); these predictive values reflect the imposed 50% stroke prevalence and should not be interpreted as population-level estimates. Agreement between GPT-5 classifications and the radiologist–neurologist consensus reference standard was almost perfect (Cohen’s κ = 0.836; χ^2^ = 158.1, df = 1, *p* < 0.001). The error patterns observed in the false-positive cases were heterogeneous and are detailed in Section 3.6; they included misinterpretation of artifactual and physiologic DWI hyperintensities, misclassification of chronic structural lesions, and unsupported report statements, all in the absence of ADC confirmation. Detailed diagnostic performance metrics and a summary of agreement across all hierarchical tasks are presented in Table 1 and Table 2, respectively.

### 3.3. Anterior Versus Posterior Circulation Classification

To further characterize localization performance, vascular territories were grouped into anterior circulation (MCA and ACA; *n* = 82) and posterior circulation (*n* = 28); posterior circulation cases in this dataset comprised exclusively PCA and PICA territory infarctions. GPT-5 correctly classified the circulatory territory in 103 of 110 cases, yielding an overall accuracy of 93.6%. Agreement between GPT-5 classifications and the radiologist–neurologist consensus reference standard was almost perfect (κ = 0.838; asymptotic standard error [ASE] = 0.059; χ^2^ = 77.6, df = 1, *p* < 0.001).

For anterior circulation cases, GPT-5 achieved a sensitivity of 93.9%, a specificity of 92.9%, and a PPV of 97.5%. For posterior circulation cases, sensitivity was 92.9% and specificity was 93.9%. Misclassifications were rare: 5 of 82 anterior circulation cases (6.1%) were misclassified as posterior, and 2 of 28 posterior circulation cases (7.1%) were misclassified as anterior. Circulation-level performance metrics are summarized in Table 3.

### 3.4. Performance in Vascular Territory Classification

Among the 110 patients with confirmed ischemic stroke, GPT-5 correctly identified the affected vascular territory (MCA, PICA, ACA, or PCA) in 100 of 110 cases, yielding an overall classification accuracy of 90.9%. Agreement between GPT-5 classifications and the radiologist–neurologist consensus reference standard was substantial (κ = 0.799; ASE = 0.058; χ^2^ = 154.4, df = 9, *p* < 0.001).

For MCA infarctions (*n* = 80), the model achieved a sensitivity of 92.5% and a PPV of 97.4%, with six cases misclassified as PCA (*n* = 3), ACA (*n* = 2), or PICA (*n* = 1). For PICA infarctions (*n* = 21), sensitivity was 95.2% and PPV was 87.0%. The ACA (*n* = 2) and PCA (*n* = 7) subgroups were too small to support reliable performance estimates and are therefore reported descriptively. The model correctly classified 1 of 2 ACA infarctions and 5 of 7 PCA infarctions. Territory-specific performance metrics with Wilson 95% confidence intervals are summarized in Table 4; ACA and PCA estimates remain descriptive because of the very small subgroup sizes.

Because the territory distribution was markedly imbalanced (MCA, *n* = 80; PICA, *n* = 21; ACA, *n* = 2; PCA, *n* = 7), class-balanced performance was additionally assessed. Macro-averaged recall, equivalent to multiclass balanced accuracy, was 77.3%, compared with an overall accuracy of 90.9%. Because each territory contributes equally to this measure, the estimate is strongly influenced by the imprecise ACA (*n* = 2) and PCA (*n* = 7) recalls. The complete four-class predicted-versus-reference confusion matrix is presented in Table 4.

### 3.5. Segment-Associated Territory Analysis of MCA Infarctions

Of the 80 radiologically confirmed MCA infarctions, GPT-5 correctly identified the MCA territory in 74 cases (92.5%). Segment-associated territory analysis was performed in these 74 cases, consistent with the sequential prompting design in which the Step 2 prompt was administered only following an MCA response. Among the 74 cases in which GPT-5 first correctly identified the MCA territory, conditional segment-associated territory accuracy was 71.6% (53/74; 95% CI, 60.5–80.6%). When all 80 reference-standard MCA cases were considered, the end-to-end hierarchical accuracy for correct MCA identification followed by correct segment-associated territory classification was 66.3% (53/80; 95% CI, 55.4–75.7%). Conditional segment-associated territory classification showed moderate agreement (κ = 0.602; ASE = 0.070; χ^2^ = 83.9, df = 9, *p* < 0.001).

Performance varied markedly across the M1–M4 segment-associated territories. The model performed best for M2, achieving a sensitivity of 92.6% (25/27), followed by M1 at 82.4% (14/17). In contrast, sensitivity was substantially lower for M3 at 37.5% (6/16), with the majority of errors (*n* = 7) representing misclassification as M2, likely reflecting the anatomical contiguity of the M2 and M3 segment-associated territories. Because the M2–M4 labels represent adjacent, partly overlapping segment-associated territories simplified into single-label categories (Section 2.4), misclassifications between neighboring territories should be interpreted in the context of this simplification. For M4, sensitivity was 57.1% (8/14), with all six misclassifications assigned to M1 (*n* = 4) or M2 (*n* = 2). Performance metrics for each segment-associated territory are presented in Table 5.

### 3.6. Error Analysis of False-Positive Cases

To characterize the error patterns in the 18 false-positive cases, all erroneous reports were evaluated for the presence of four non-mutually exclusive and non-exhaustive error categories. Multiple categories could be assigned to a single case. At least one category was assigned to 16 of 18 reports (88.9%); in the remaining two reports, the diagnosis was explicitly conditioned on unavailable ADC confirmation, and no category criterion was met (Supplementary Table S1).

Artifactual and Physiologic Signal Misinterpretation. This category was identified in 13 of 18 false-positive cases (72.2%). In 12 cases, posterior fossa hyperintensity, with or without occipital lobe involvement, was misidentified as acute posterior circulation infarction. In two cases, physiologic diffusion anisotropy in the corpus callosum splenium (*n* = 1) or the bilateral thalami (*n* = 1) was reported as pathological restricted diffusion; one case exhibited both patterns, yielding 13 distinct cases in this category.

Chronic Lesion Misclassification. Five cases (27.8%) involved misclassification of chronic structural changes as acute ischemic lesions. Periventricular white matter hyperintensities consistent with leukoaraiosis, dilated perivascular spaces in the deep hemispheric regions, and chronic watershed microangiopathy were interpreted as new infarctions.

Terminological Inconsistency or Unsupported Report Statements. This category was identified in 14 of 18 cases (77.8%). The term “restricted diffusion”—which requires ADC confirmation—was applied on the basis of DWI signal alone in 14 reports (77.8%), with a terminological inconsistency arising when confirmatory sequences were unavailable. Additionally, six reports (33.3%) contained statements evaluating hemorrhage exclusion, mass effect, ventricular size, and midline shift from DWI alone—features that cannot be assessed on isolated DWI (see Section 4.2).

Etiological Scenarios or Unhedged Definitive Diagnoses. This category was identified in 7 of 18 cases (38.9%). The etiological scenarios generated for artifactual or physiologic signal included multifocal embolic stroke (*n* = 2), hemodynamic compromise with watershed infarction (*n* = 1), and a top-of-the-basilar occlusion pattern (*n* = 1). In three additional cases, definitive diagnostic language was used without hedging despite the absence of ADC confirmation.

In all 15 false-positive reports that assigned a definite vascular territory (15 of 18; 83.3%), the named territory was anatomically concordant with the location of the signal abnormality described in the report; the remaining three reports did not assign a definite territory.

### 3.7. Exploratory Response-Stability Analysis

In the separate 34-case prompt-development image set (Section 2.3), GPT-5 classified 21 cases as stroke in the first run and 19 in the second. Binary stroke classification was concordant in 32 of 34 cases (94.1%; 95% CI, 80.9–98.4%; Cohen’s κ = 0.879; ASE = 0.082; *p* < 0.001); both discordant cases were classified as stroke in the first run and as no stroke in the second. Among the 19 cases classified as stroke in both runs, vascular territory assignment was concordant in 18 (94.7%; 95% CI, 75.4–99.1%).

## 4. Discussion

### 4.1. Overall Diagnostic Performance

In this study, GPT-5 demonstrated substantial diagnostic capability in classifying preselected representative DWI images across three levels of increasing anatomical granularity. For binary stroke detection, agreement with the radiologist–neurologist consensus reference standard was almost perfect (κ = 0.836), with an observed sensitivity of 100% and a specificity of 83.6%. For vascular territory classification, agreement remained almost perfect at the circulatory level (κ = 0.838) and substantial at the territory level (κ = 0.799). Performance declined to moderate agreement for MCA segment-associated territory classification (κ = 0.602), with a conditional accuracy of 71.6% (53/74) and an end-to-end hierarchical accuracy of 66.3% (53/80).

This stepwise decline in performance—from binary stroke detection to increasingly detailed anatomical localization—is clinically plausible and consistent with previous studies showing that LLMs perform more reliably on high-level classification tasks than on fine-grained anatomical reasoning [6,7]. However, this pattern should be interpreted in the context of the limited interpretability of LLMs. Because their internal decision-making processes are not directly observable—the so-called “black box” problem [10,11]—the mechanisms underlying both correct and incorrect predictions remain difficult to explain.

### 4.2. False-Positive Error Analysis and Model Limitations

The four error categories identified among the 18 false-positive cases illustrate important limitations of applying general-purpose LLMs to specialized neuroimaging tasks. Detailed category definitions, frequencies, and representative examples are provided in Table 6; the following discussion focuses on the potential mechanisms and clinical implications of these error patterns rather than restating category frequencies.

Artifactual and physiologic signal misinterpretation, a leading category, is consistent with motion artifacts, image quality variability, and non-ischemic signal abnormalities previously described as fundamental sources of false-positive output in AI-based stroke detection [4,12]. The model systematically misidentified posterior fossa susceptibility and pulsation artifacts as acute posterior circulation infarctions—an error pattern consistent with well-established technical limitations of echo-planar DWI acquisition in this region [13,14,15], and potentially reflecting an underrepresentation of DWI-specific imaging physics in the model’s training. The absence of ADC confirmation is particularly relevant to this error pattern, because true diffusion restriction could not be confirmed and alternative sources of DWI hyperintensity, including T2 shine-through, could not be excluded; susceptibility- and pulsation-related abnormalities were also prominent among the observed false-positive cases.

Chronic lesion misclassification may reflect the model’s limited temporal discrimination in the absence of clinical context. Age-related periventricular leukoaraiosis, dilated perivascular spaces [16,17,18], and chronic small-vessel disease were interpreted as acute infarctions, consistent with a challenge also documented in deep-learning models for DWI-based stroke detection [19,20].

Terminological inconsistency or unsupported report statements, the most frequent category overall, encompassed two related behaviors. First, the term “restricted diffusion”—a radiological designation requiring multimodal (ADC) confirmation—was applied on the basis of DWI signal alone, a systematic terminological inconsistency arising when confirmatory sequences were unavailable. Second, and more revealing, although the prompt explicitly requested output formatted as a radiological report, the inclusion of statements regarding hemorrhage, mass effect, ventricular size, and midline shift in a subset of reports (*n* = 6) represents a clinically significant failure mode rather than a benign formatting artifact. These features cannot be assessed on isolated DWI, and their unqualified inclusion would be unsafe if acted upon without oversight. Their appearance is consistent with clinically ungrounded template completion: because these features are not evaluable on the provided images, statements affirming or excluding them cannot derive from image analysis and may instead reflect reproduction of expected report content. This behavior, which may have been related to the requested report format, represents a clinically important safety risk because the model may generate apparently authoritative statements about findings that are not assessable from the available images. This finding reinforces that general-purpose LLMs should not be used for independent image interpretation without expert oversight [21].

Etiological scenarios or unhedged definitive diagnoses may represent the most clinically relevant category. Reports in this category contained either etiological scenarios for artifactual or physiologic signal or unhedged definitive diagnoses, despite the absence of ADC confirmation. The scenario-based reports were internally coherent yet clinically implausible, a pattern consistent with the confabulation phenomenon described in the medical AI literature [11,22]. In an emergency workflow, such apparently coherent false-positive interpretations could, if acted upon without expert verification, prompt unnecessary downstream imaging, divert attention from or delay consideration of alternative diagnoses, or lead clinicians to consider acute stroke interventions that are not otherwise supported by the full clinical and imaging context.

The anatomical concordance observed in these 15 false-positive reports (Section 3.6) indicates internal consistency between the location described by the model and its vascular territory labeling, but does not represent validated localization accuracy in the absence of true infarction. Taken together with the error-category distribution, the false-positive failures observed in this cohort were characterized mainly by misclassification of artifactual, physiologic, or chronic signal and by unsupported or overconfident report statements, rather than by discordance between the described location and the assigned vascular territory.

### 4.3. Clinical Implications

The high sensitivity observed under the experimental conditions of this study suggests potential utility for GPT-5 in preliminary image-based diagnostic stratification. This framework may also resemble a clinician-assisted use case in which a physician submits selected suspicious DWI images for secondary AI interpretation. However, because slice selection in the present study was performed with knowledge of the reference-standard case status, this potential workflow requires prospective validation using a prespecified image-selection protocol applied before reference-standard adjudication. In addition, the 16.4% false-positive rate, particularly in the context of etiological scenarios or unhedged definitive diagnoses, underscores the continued need for expert interpretation. These findings support further evaluation of GPT-5 as a potential clinician-assisted image-interpretation tool but do not establish a clinical decision-support role.

The balanced case–control design imposed a 50% stroke prevalence. Accordingly, the cohort-specific PPV and NPV should not be extrapolated to unselected emergency department populations. At lower disease prevalence, the same false-positive rate would result in a lower PPV and a greater false-positive burden relative to true-positive detections.

The generation of unsupported statements about hemorrhage, mass effect, ventricular size, and midline shift further illustrates why expert oversight is essential when general-purpose LLMs are applied to neuroimaging. As discussed in Section 4.2, such false-positive outputs could also have downstream consequences in emergency care if accepted without expert verification. These risks also underscore the need for prospective evaluation within real clinical workflows.

These findings also reinforce the need for standardized, validated prompting frameworks before clinical deployment. Without such standardization, LLM-based image interpretation risks producing highly variable outputs across institutions, potentially introducing new sources of diagnostic error.

### 4.4. Comparison with Prior Literature

Previous studies evaluating general-purpose LLMs for acute ischemic stroke detection have used different model generations, imaging inputs, prompting strategies, eligibility criteria, and study populations [3,5]. Koyun and Taskent evaluated ChatGPT-4o and Claude 3.5 Sonnet using paired DWI–ADC images with a binary diagnostic prompt [3], whereas the present study evaluated GPT-5 using preselected DWI images and a structured vascular territory-based prompting framework. ChatGPT-4o showed 100% sensitivity and 3.6% specificity in that study, while GPT-5 showed 100% sensitivity and 83.6% specificity in the present cohort. Because these estimates were obtained under substantially different experimental conditions, the numerical differences should not be interpreted as evidence of superiority of one model or prompting strategy over another.

Kuzan et al. reported 79.6% sensitivity and 84.9% specificity for ChatGPT-4V using combined DWI and ADC images [5]. Their eligibility criteria, imaging inputs, and case composition differed from those of the present study, including exclusion of lacunar infarcts smaller than 1 cm. Accordingly, the similar specificity estimates observed across the two studies should be regarded as descriptive cross-study observations rather than directly comparable performance measures.

An additional distinction from previous studies concerns the framework used for lesion localization. Earlier investigations assessed localization using hemispheric or lobar anatomy, reporting localization accuracies ranging from 7.3% to 32.7% for ChatGPT models and a hemispheric accuracy of 67.2% for Claude 3.5 Sonnet [3,5]. In contrast, we evaluated localization according to arterial vascular territories, achieving accuracies of 93.6% at the circulatory level and 90.9% at the individual vascular territory level. Because these approaches represent different diagnostic frameworks, direct numerical comparisons should be interpreted cautiously. Vascular territory-based localization represents a distinct anatomical framework that may be clinically relevant for DWI interpretation; however, its relative performance compared with lobar or hemispheric localization cannot be determined from cross-study comparisons. Territory-specific interpretation is further limited by the very small ACA and PCA subgroups, for which the present data are descriptive rather than generalizable. Future studies comparing vascular, lobar, and hemispheric localization strategies within the same model would help clarify the independent contribution of prompt design.

Finally, comparisons with dedicated deep-learning models should be interpreted with caution. Such systems are typically trained on multimodal datasets and optimized for lesion-level detection or segmentation [4,23], whereas the present study evaluated a DWI-only LLM in a zero-shot setting, that is, without any task-specific training or fine-tuning performed by the investigators. These approaches thus address different clinical and technical objectives. General-purpose LLMs represent a distinct approach from dedicated imaging models, but their potential clinical utility and workflow integration require direct prospective evaluation.

### 4.5. Limitations

Several limitations warrant consideration. First, this was a retrospective, single-center study, which may limit the generalizability of the findings to other institutions, scanner protocols, patient populations, and emergency department workflows. Prospective multicenter validation will be necessary before any clinical deployment can be considered. Second, the balanced case–control design imposed a 50% stroke prevalence and does not reflect the prevalence encountered in unselected emergency department populations. Consequently, the reported PPV and NPV are cohort-specific and should not be interpreted as real-world predictive values. At lower stroke prevalence, the observed 16.4% false-positive rate would yield a substantially lower PPV and a greater burden of false-positive alerts. For illustration, applying the observed sensitivity and specificity to the full eligible study population (110 stroke-positive among 990 examinations; prevalence 11.1%) would correspond to approximately 144 false-positive outputs for 110 true-positive outputs, i.e., a PPV of approximately 43%. Third, ADC maps were deliberately withheld to evaluate GPT-5 under isolated-DWI conditions. This represents an artificial diagnostic setting compared with routine clinical practice, in which DWI is interpreted together with ADC to confirm true diffusion restriction and distinguish it from T2 shine-through and susceptibility- or pulsation-related artifacts. The absence of ADC confirmation may therefore have contributed to the observed false-positive error profile, and performance under standard DWI–ADC evaluation may differ. By contrast, the reference-standard panel had access to the complete multimodal MRI examination and presenting clinical findings; this information asymmetry may have influenced the observed error profile. Fourth, the very small ACA (*n* = 2) and PCA (*n* = 7) subgroups preclude reliable territory-specific performance estimates and limit generalizability beyond the more highly represented MCA and PICA territories. Findings for ACA and PCA should therefore be considered descriptive only. Posterior circulation cases were also limited to PCA and PICA infarctions; no basilar artery or superior cerebellar artery territory infarctions and no brainstem infarctions were represented, further limiting generalizability across posterior circulation stroke. Fifth, slice selection was performed after reference-standard classification, and the two emergency physicians were therefore aware of case status during image selection; moreover, stroke-positive and control cases were subject to different selection rules (maximum signal abnormality versus anatomical level). In stroke-positive cases, selecting slices showing the maximum signal abnormality may have introduced image-selection and spectrum effects and simplified the diagnostic task. Accordingly, the reported performance should be interpreted as classification and localization performance on curated representative images rather than autonomous detection across complete DWI examinations. Sixth, DICOM-to-JPEG conversion for AI input may have altered subtle image features, potentially affecting model performance; native DICOM-based evaluation may yield different results. Seventh, GPT-5 Thinking was evaluated through the ChatGPT interface, where backend version consistency could not be independently verified. Platform and model updates may therefore limit exact reproducibility and the applicability of the findings to subsequent versions. In addition, because GPT-5 is a proprietary model trained on undisclosed data, prior exposure to similar cases or images during model training cannot be excluded. Eighth, no human-reader comparator (e.g., emergency physicians or radiologists interpreting the same preselected images under identical conditions) was included; therefore, the incremental value of GPT-5 relative to current clinical practice cannot be inferred from the present data.

## 5. Conclusions

GPT-5 demonstrated substantial diagnostic performance in classifying preselected representative DWI images for acute ischemic stroke using a vascular territory-based framework, with accuracy declining at progressively finer levels of anatomical localization. The observed 100% sensitivity was accompanied by a 16.4% false-positive rate, with false-positive outputs characterized mainly by misclassification of artifactual, physiologic, or chronic signal and by unsupported or overconfident report statements. These findings support further evaluation of GPT-5 as a potential clinician-assisted image-interpretation tool but do not establish a clinical decision-support role. Its potential role in preliminary image-based diagnostic stratification should be approached cautiously and prospectively validated in clinically representative populations, with continued expert interpretation.

## Figures and Tables

**Table 1:** Diagnostic performance of GPT-5 for stroke detection on DWI (*n* = 220).

Metric	Value	95% CI
Sensitivity	100%	96.6–100%
Specificity	83.6%	75.6–89.4%
PPV	85.9%	78.9–90.9%
NPV	100%	96.0–100%
Accuracy	91.8%	87.4–94.8%
Cohen’s κ	0.836	—

Abbreviations: CI, confidence interval; PPV, positive predictive value; NPV, negative predictive value; DWI, diffusion-weighted imaging; TP, true positive; FN, false negative; FP, false positive; TN, true negative. The underlying 2 × 2 classification counts were TP = 110, FN = 0, FP = 18, and TN = 92. Because of the balanced case–control design, PPV and NPV are cohort-specific values based on the imposed 50% stroke prevalence and should not be interpreted as estimates for an unselected emergency department population.

**Table 2:** Summary of diagnostic agreement across all hierarchical tasks.

Diagnostic Task	*n*	Accuracy	Cohen’s κ	Agreement Level
Stroke detection (binary)	220	91.8%	0.836	Almost perfect
Anterior/posterior circulation	110	93.6%	0.838	Almost perfect
Vascular territory (4 territories)	110	90.9%	0.799	Substantial
MCA segment-associated territory (M1–M4), conditional	74	71.6%	0.602	Moderate

Agreement interpretation criteria: 0.41–0.60 = moderate; 0.61–0.80 = substantial; 0.81–1.00 = almost perfect.

**Table 3:** Anterior versus posterior circulation classification performance of GPT-5 (*n* = 110).

Circulation	*n* (Ref.)	Correct	Sensitivity (95% CI)	Specificity (95% CI)	PPV (95% CI)	NPV (95% CI)
Anterior (MCA + ACA)	82	77	93.9% (86.5–97.4)	92.9% (77.4–98.0)	97.5% (91.2–99.3)	83.9% (67.4–92.9)
Posterior (PCA + PICA)	28	26	92.9% (77.4–98.0)	93.9% (86.5–97.4)	83.9% (67.4–92.9)	97.5% (91.2–99.3)

Posterior circulation cases included PCA and PICA territory infarctions only. Confidence intervals are Wilson 95% confidence intervals. Ref., reference standard; MCA, middle cerebral artery; ACA, anterior cerebral artery; PCA, posterior cerebral artery; PICA, posterior inferior cerebellar artery; PPV, positive predictive value; NPV, negative predictive value. No basilar artery, superior cerebellar artery, or brainstem infarctions were represented.

**Table 4:** Vascular territory classification performance of GPT-5 (*n* = 110).

*(a) Complete predicted-versus-reference confusion matrix*					
**Reference Territory ↓/GPT-5 Prediction →**	**MCA**	**PICA**	**ACA**	**PCA**	**Total**
MCA	74	1	2	3	80
PICA	1	20	0	0	21
ACA	0	1	1	0	2
PCA	1	1	0	5	7
Predicted total	76	23	3	8	110
*(b) Territory-specific one-versus-rest performance estimates*					
**Territory**	**Reference *n***	**Sensitivity** **(95% CI)**	**Specificity** **(95% CI)**	**PPV** **(95% CI)**	**NPV** **(95% CI)**
MCA	80	92.5% (84.6–96.5)	93.3% (78.7–98.2)	97.4% (90.9–99.3)	82.4% (66.5–91.7)
PICA	21	95.2% (77.3–99.2)	96.6% (90.6–98.8)	87.0% (67.9–95.5)	98.9% (93.8–99.8)
ACA	2	50.0% (9.5–90.5)	98.1% (93.5–99.5)	33.3% (6.1–79.2)	99.1% (94.9–99.8)
PCA	7	71.4% (35.9–91.8)	97.1% (91.8–99.0)	62.5% (30.6–86.3)	98.0% (93.1–99.5)

Overall territory classification accuracy was 100/110 (90.9%); macro-averaged recall, equivalent to multiclass balanced accuracy, was 77.3%. In part (a), rows (↓) show the reference-standard territory and columns (→) the GPT-5 prediction. Confidence intervals are Wilson 95% confidence intervals. ACA and PCA estimates are imprecise because of the very small reference-standard subgroup sizes and should be interpreted descriptively. MCA, middle cerebral artery; PICA, posterior inferior cerebellar artery; ACA, anterior cerebral artery; PCA, posterior cerebral artery.

**Table 5:** Conditional MCA segment-associated territory classification performance among cases in which GPT-5 first correctly identified the MCA territory (*n* = 74).

Segment-Associated Territory	*n*	Correct	Sensitivity (95% CI)	FN	Misclassified As
M1	17	14	82.4% (59.0–93.8)	3	M2 (*n* = 1), M3 (*n* = 1), M4 (*n* = 1)
M2	27	25	92.6% (76.6–97.9)	2	M1 (*n* = 1), M3 (*n* = 1)
M3	16	6	37.5% (18.5–61.4)	10	M2 (*n* = 7), M4 (*n* = 2), M1 (*n* = 1)
M4	14	8	57.1% (32.6–78.6)	6	M1 (*n* = 4), M2 (*n* = 2)

Segment-associated territory analysis was restricted to the 74 cases in which the MCA territory identified by GPT-5 was confirmed by the reference standard; cases assigned to the MCA territory by GPT-5 but not by the reference standard had no reference segment-associated territory label. Conditional segment-associated territory accuracy among the 74 cases in which GPT-5 first correctly identified the MCA territory was 53/74 (71.6%; 95% CI, 60.5–80.6%). Across all 80 reference-standard MCA cases, end-to-end hierarchical accuracy for correct MCA identification followed by correct segment-associated territory classification was 53/80 (66.3%; 95% CI, 55.4–75.7%). Confidence intervals are Wilson 95% confidence intervals. FN, false negative.

**Table 6:** Error categories identified in the 18 false-positive cases.

Error Category	*n* (%)	Coded Features	Representative Example
Artifactual and Physiologic Signal Misinterpretation	13 (72.2%)	Posterior fossa susceptibility/pulsation artifacts (*n* = 12) and physiologic anisotropy in the splenium or bilateral thalami (*n* = 2); one case showed both patterns.	“Restricted diffusion is observed in multiple regions, notably in the cerebellar hemispheres, as well as in the occipital and parietal lobes.” (FP-05)
Chronic Lesion Misclassification	5 (27.8%)	Age-related periventricular white-matter change, dilated perivascular spaces, and chronic small-vessel/watershed change misclassified as acute ischemia.	“Small focal diffusion hyperintense lesion in the left basal ganglia/corona radiata region… consistent with a small acute/subacute ischemic infarct.” (FP-04)
Terminological Inconsistency or Unsupported Report Statements	14 (77.8%)	Use of “restricted diffusion” on DWI alone (*n* = 14); unsupported statements on hemorrhage, ventricular size, mass effect, or midline shift (*n* = 6).	“No clear evidence of hemorrhage, mass effect, or midline shift is observed. Ventricular system appears normal.” (FP-06)
Etiological Scenarios or Unhedged Definitive Diagnoses	7 (38.9%)	Etiological scenarios (multifocal embolism, hemodynamic/watershed, or top-of-the-basilar pattern) reported for artifactual or physiologic signal (*n* = 4); unhedged definitive diagnostic language (*n* = 3).	“The pattern of involvement may indicate a multifocal embolic process, watershed infarcts, or a posterior circulation stroke.” (FP-15)

Categories were non-mutually exclusive and non-exhaustive. Two emergency physicians independently coded all 18 false-positive cases using the predefined coding framework; discrepant assignments were reviewed with the radiologist and neurologist and resolved by consensus. Anonymized case-level coding and sub-code assignments are provided in Supplementary Tables S1 and S2, with operational definitions in Supplementary Methods. DWI, diffusion-weighted imaging; ADC, apparent diffusion coefficient.

## Data Availability

The data presented in this study are available from the corresponding author upon reasonable request. As the data consist of patient-derived diffusion-weighted MRI images, any data sharing is subject to prior approval by the Non-Interventional Clinical Research Ethics Committee of Yozgat Bozok University and the execution of a data-sharing agreement, in accordance with patient privacy and ethical restrictions.

## Supplementary Materials

The following supporting information can be downloaded at https://www.mdpi.com/article/10.3390/jcm15197716/s1, Table S1: Case-level false-positive error coding; Table S2: Sub-code assignments underlying the category frequencies in Table 6. Supplementary Methods: False-positive error coding protocol.
